# Carbon Nanomaterials for the Treatment of Heavy Metal-Contaminated Water and Environmental Remediation

**DOI:** 10.1186/s11671-019-3167-8

**Published:** 2019-11-11

**Authors:** Rabia Baby, Bullo Saifullah, Mohd Zobir Hussein

**Affiliations:** 1 Education Department Sukkur IBA University, Sukkur, Sindh 65200 Pakistan; 2 MSCL, Institute of Advanced Technology, University Putra Malaysia, 43400 Serdang, Selangor Malaysia

**Keywords:** Carbon nanotubes (CNTs), Multiwall carbon nanotubes (MWCNTs), Single-wall carbon nanotubes (SWCNTs), Fullerene, Graphene, Graphene oxide (GO), Activated carbon (AC), Heavy metals, Water purification

## Abstract

Nanotechnology is an advanced field of science having the ability to solve the variety of environmental challenges by controlling the size and shape of the materials at a nanoscale. Carbon nanomaterials are unique because of their nontoxic nature, high surface area, easier biodegradation, and particularly useful environmental remediation. Heavy metal contamination in water is a major problem and poses a great risk to human health. Carbon nanomaterials are getting more and more attention due to their superior physicochemical properties that can be exploited for advanced treatment of heavy metal-contaminated water. Carbon nanomaterials namely carbon nanotubes, fullerenes, graphene, graphene oxide, and activated carbon have great potential for removal of heavy metals from water because of their large surface area, nanoscale size, and availability of different functionalities and they are easier to be chemically modified and recycled. In this article, we have reviewed the recent advancements in the applications of these carbon nanomaterials in the treatment of heavy metal-contaminated water and have also highlighted their application in environmental remediation. Toxicological aspects of carbon-based nanomaterials have also been discussed.

## Introduction

**Fig. 1:**
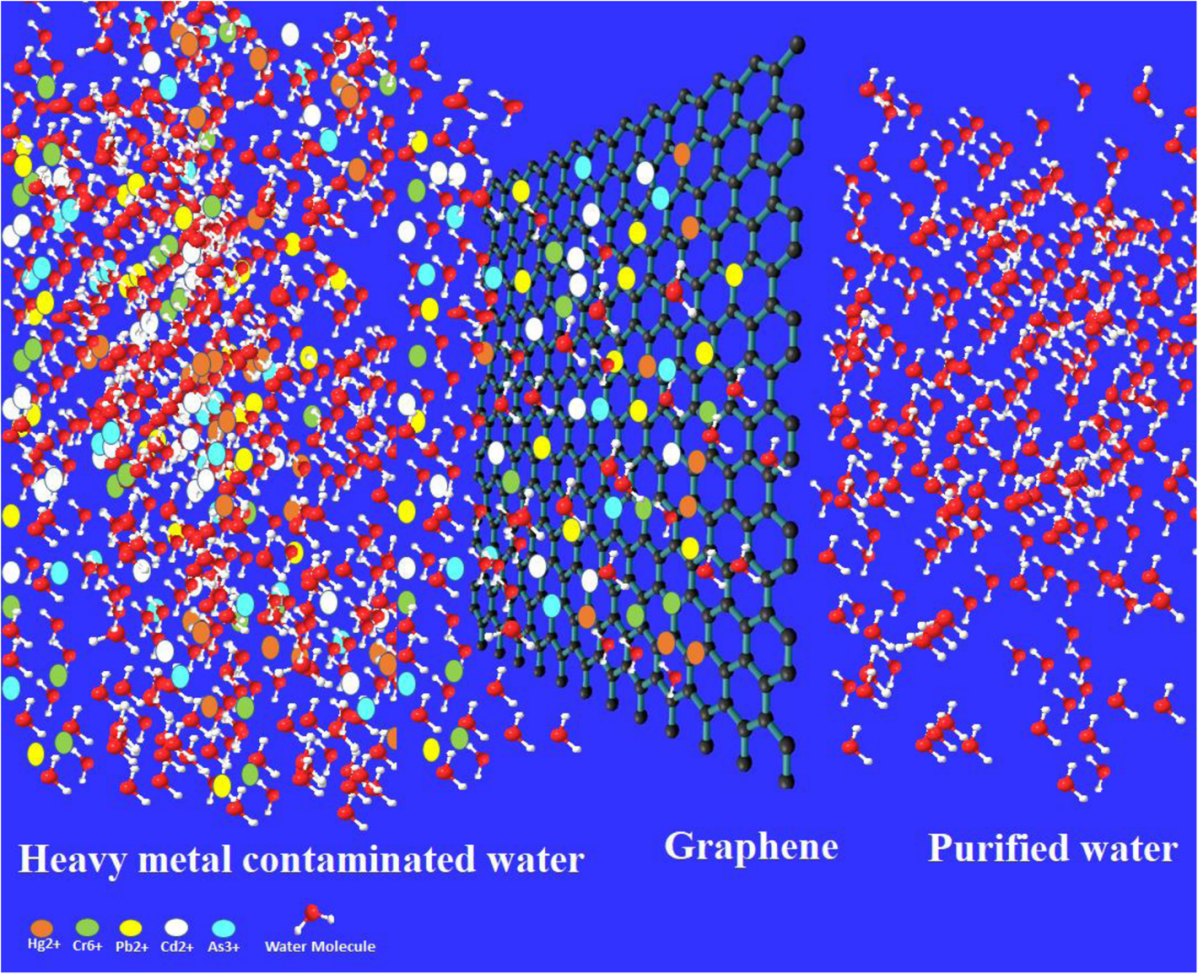
Graphical abstract showing that the heavy metal-contaminated water purification process using graphene and other carbon-based materials can also do the same

**Fig. 2:**
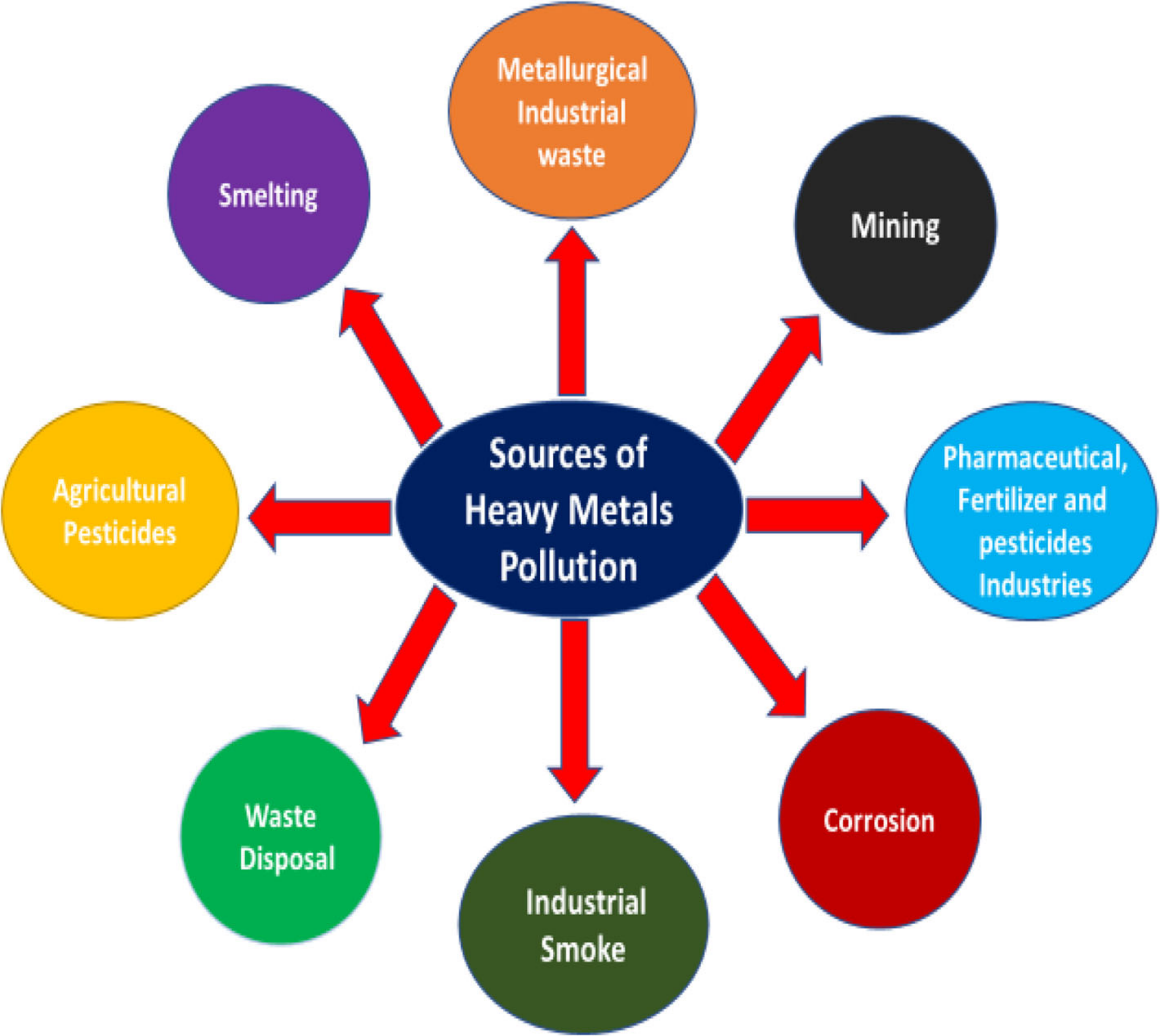
Sources of heavy metal contamination in the environment

Pollution is termed as the presence of undesirable chemical entity/entities preventing the natural process or causing adverse effects to living organisms and the environment [1–3]. Industrialization and immense increase in population leading to growing urbanization cause the increase in pollution at an alarming rate [2, 4]. Improving the water, soil, and air quality is an immense challenge of the modern era. Identification and treatment of environmental pollutants and their prevention is a key step in the protection of the environment. Material science plays a vital role in realizing the clean environmental goal, and materials science technology has progressed exponentially in the last decade especially nanomaterials [1, 5]. The pure and clean water is getting scarce due to industrialization, and the world is facing a shortage of clean water especially in the developing world [6]. Water contaminants can be organics, bacteria, viruses, dyes, and heavy metal ions such as lead, cadmium, zinc, nickel, arsenic, chromium, and mercury with nonbiodegradable nature posing a great risk to human health. Heavy metal ions can cause many adverse effects like cancer, kidney damage, hepatitis, miscarriages, anemia, encephalopathy, and nephritic syndrome [7–10]. Lead ions are released in the environment generally from metal mining industries of acid lead batteries, paper, glass, and polishing industries. Cadmium is generally found in water discharged from electroplating designing of batteries, photovoltaic cell, metallurgy process, and fabric factories [11]. Nickel ions can cause skin diseases when contacted with jewelry trashing, zips, watches, coins, etc. Chromium metal ions (VI) cause diseases like liver damage, nephritis, and stomach distresses, and Cr (VI) ions are also the major cause of nasal mucous ulcer [12]. Figure 1 shows the adsorption of heavy metal ions on the carbon nanomaterial (graphene), and Fig. 2 highlights the different sources of heavy metal contamination in the environment. Because of these severe adverse effects, removal of heavy metal ions from water is of prime importance for saving the human lives from such problematic health issues. Toxic metal ions could be removed by numerous methods, like ion exchange, reverse osmosis, precipitation filtration, biosorption, coagulation, and extraction [13, 14]. Adsorption is considered as the best method as it is cost-effective, highly efficient, and easy to operate for removing trace levels of heavy metal ions [15]. Different materials have been applied for water treatment such as plant adsorbents and organic nature materials especially humic acid which has been widely applied for water disinfection and for the removal of heavy metal ions [8, 16–19]. Wang et al. have comprehensively reviewed the humic acid and its nanocomposite in water treatment [20].

Nanotechnology platform finds application almost in every field such as environmental science, health sciences, electronics, industrial separation, portable water treatment large/small scale plants, catalyst, energy storage, and energy generation [21–23]. Nanomaterials provide a special platform for the purification of contaminated water due to the high surface area of nanosorbents and their capability of chemical modification and easier regeneration. Nanomaterials are being exploited more and more for the removal of different types of pollutants namely organics, metal ions, biological contaminants, and arsenic from the water [24–27]. Carbon nanomaterials namely graphene, graphene oxide, carbon nanotubes, fullerenes, and activated carbons have been widely used in energy storage, sensors, electronics, water purification, drug delivery, disease diagnosis, etc. owing to their exceptional chemical, mechanical, thermal, and electrical characteristics. In this article, we have tried to review the latest advancement in the application of carbon nanomaterials namely fullerenes, carbon nanotubes (CNTs), graphene, graphene oxide, and activated carbon in purification heavy metal ion-contaminated water.

## Human Health and Heavy Metal Toxicity

The heavy metals are usually defined based on their atomic weights; however, term heavy metals are also referred to elements toxic to living creatures [28]. Certain heavy metals are lethal to the human health and other living creatures in their different forms and doses (Table 1). Frequently, heavy metals are thought of as toxic; however, lighter metals may likewise be lethal, for example, beryllium and lithium. Not all heavy metals are poisonous to health, as some are fundamental, for example, iron and Cr (III). Most commonly known toxic metals are Pb, Cd, Cr, Mn, Hg, As, and radioactive metals. Radioactive metals have both radiological and chemical toxicity. Heavy metal toxicity has turned out to be a major threat, and there are many health risks associated with them. The lethal impact of these metals is the fact that they do not have any biological role; however, they may mimic as an element of the body and interfere with the metabolic and other biological processes. Some metals like aluminum can easily be eliminated by the excretion system, while certain metals accumulated in the food chain and body. Metal-induced toxicity depends on dose, exposure route, and contact time (e.g., acute or chronic). Details of toxicity associated with different heavy metals are given below.

**Table 1:** Adverse effect of heavy metals on human health

S. No	Metal ion	Adverse effects on human health
1	Hg^1+^	Liver damage, neural damage, gastrointestinal toxicity, neurotoxicity, and nephrotoxicity [29–31]
2	Pb^2+^	Neural damage, kidney damage, gastrointestinal diseases, decrease in male sperm count in men, and abortions in women [33, 34]
3	Cd^2+^	Kidney failure, bone disorders, chronic anemia, and cancer of the prostate, kidney, hematopoietic system, liver, and stomach [37, 38]
4	Cr^6+^	Cancer of respiratory tract and stomach, and liver damage [39, 40]
5	As^3+^	Cancer, liver and kidney failure, respiratory problems, diabetes, miscarriages, neural damage, and cardiovascular problems [42–44]
6	Zn^2+^	Stomach cramps, fever, vomiting, nausea, and diarrhea [49]

### Toxicity of Mercury (Hg)

Mercury (Hg) is a d-block element with an atomic number 80 and is in liquid form under standard conditions. Mercury is found in deposits of mercuric sulfide called cinnabar. Mercury pollution is caused by pharmaceutical industries, pulp and paper preservation, caustic soda production industry, agriculture industry, etc. [47]. Mercury is the most toxic heavy metal in the environment, and mercury poisoning is called pink disease also known acrodynia. Mercury can combine with organic and inorganic compounds. Elevated exposure levels of mercury in any form can damage the kidneys, brain, developing fetus, etc. [48]. The environmental protection agency has declared methyl mercury and mercuric chloride as carcinogenic. Mercury exposure can also cause lung damage, skin rashes, memory problems, and hair loss. The World Health Organization (WHO) has set the standard for drinking water with lower levels of mercury to 0.01 mg/l [29].

### Toxicity of Lead (Pb)

Lead (Pb) is an element with an atomic number of 82 and is considered as a heavy metal with silvery bluish appearance which turns dull gray by the action of air [30]. There are various sources of lead pollution, mainly wastes of battery industries, fertilizers and pesticides, metal plating and finishing operations, exhaust, additives in gasoline, pigment in automobiles, and smelting of ores. This heavy toxic metal is becoming an environmental and health concern around the globe due to its widespread use [31]. Lead (Pb) is a carcinogenic element declared by the Environmental Protection Agency (EPA). Lead poisoning is a term used for its toxicity, and it may be acute or chronic. Lead poisoning can cause mental retardation, birth defects like autism, allergies, dyslexia, paralysis, brain damage, and kidney damage and may also result in death [32].

### Toxicity of Arsenic (As)

Arsenic is a metalloid element having an atomic number of 33 and occurs in mineral form commonly in combination with sulfur, some other metals, salts of iron, calcium, sodium, and copper, and also in pure elemental form [33]. The water is contaminated by arsenic-based pesticides, deposits of natural minerals, and inappropriate disposal of arsenic-based reagents or chemicals. Arsenic in the form of arsenate and arsenite is lethal to the environment and living creatures. Arsenic disturbs protoplasm of the cells by interacting with the sulphydryl group of the cells causing respiration malfunctioning and affecting mitosis and cell enzymes [34].

### Toxicity of the Cadmium (Cd)

Cadmium has an atomic number of 48 and is bluish-white soft metal having chemical properties similar to mercury and zinc of group 12 [30]. They are being produced from smelting of its ores, electroplating, batteries, plasticizers, alloys, pigments, nuclear industry, and cigarette smoke. Generally, cadmium is present at low levels in the environment; however, industrial wastes have greatly increased those levels. Cadmium-induced toxicity can cause damage to the kidneys, respiratory systems, and skeleton and is carcinogenic to humans [30, 33]. Cadmium is ranked the seventh most toxic metal by the Agency for Toxic Substances and Disease Registry (ATSDR) [34].

### Toxicity of Chromium (Cr)

Chromium (Cr) is an element having an atomic number 24, with steely gray appearance [35]. Chromium occurs in different states, e.g., divalent, tetravalent, pentavalent, and hexavalent states; however, trivalent and hexavalent forms are the most stable. Chromium (III) is an essential nutritional supplement for humans and animals [35]. However, chromium (VI) form is highly toxic and carcinogenic in nature [36, 37]. Chromium is produced in environment matrices (air, water, and soil) from different sources, e.g., wastewater and air mainly released from metallurgical and chemical industries. The hexavalent chromium Cr (VI) is an industrial pollutant established as a human carcinogen [38, 39]. Concentration of Cr (VI) in ground water and surface water is exceeding and the World Health Organization (WHO) has set the limit of 50 μg per liter [40].

### Toxicity of Zinc (Zn)

Zinc (Zn) is an element having an atomic number 30 and placed in group 2 of the periodic table. Although zinc is an essential trace metal for humans, excessive absorption of zinc can suppress the iron absorption. Zinc ions are highly toxic to plants, vertebrate fishes, invertebrates, etc. [41–43].

## Classification of Carbon Nanomaterials Based on their Dimensions

**Fig. 3:**
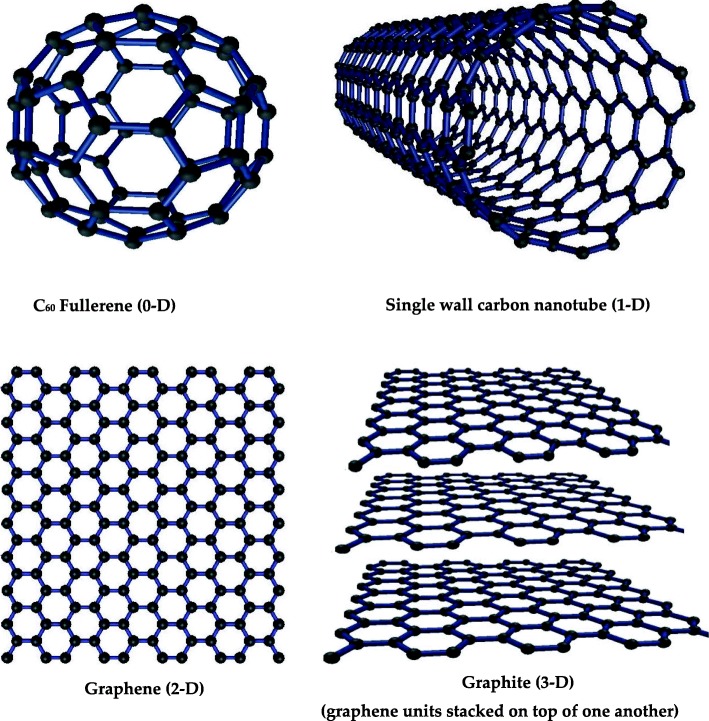
The examples of carbon nanomaterial of different dimensions

The nanomaterials having all the three dimensions less than 100 nm are termed as zero-dimensional (0-D) nanomaterial; examples are fullerene and quantum dots [44]. The nanomaterials having only one dimension larger than 100 nm and two dimensions smaller than 100 nm are termed as one-dimensional (1-D) nanomaterials, e.g., nanotubes of carbon and titanium [45, 46]. The nanomaterials whose two dimensions are greater than 100 nm are termed as two-dimensional nanomaterials, a famous example is graphene. Three-dimensional materials whose all dimensions are greater than 100 nm are termed as three-dimensional (3-D) materials; examples are graphite and some composites of nanomaterials [46]. Figure 3 shows some representative famous structure carbon materials with different dimensions, e.g., fullerene 0-D, single-wall carbon nanotube 1-D, graphene 2-D, and graphite 3-D.

### Application of Fullerenes in Environmental Remediation and Water Purification

Fullerenes were discovered in 1985 from interstellar dust, and they have a closed-caged pentagonal and hexagonal ring structure, represented with the formula of C_20 + *m*_ where *m* is an integer [49]. They have a hydrophobic character, high electron affinity and high surface to volume ratio, and surface defects. These unique physicochemical properties make them an ideal material for various applications such as semiconductors, electronics, biomedical sciences, solar cells, sensors, cosmetics, artificial photosynthesis, and surface coatings [50–52]. Brunet et al. showed that hydrophilic functionalized fullerenes (C_60_) have also been applied for the killing of the pathogenic microorganisms in water by utilizing a photocatalytic process. Fullerenes are also ideal clean green materials for hydrogen storage as fullerene molecules can easily be converted to C–C bonds to C–H because of the lower bond energies of the carbon and hydrogen [27]. The fullerenes have been reported to have the maximum storage 6.1% hydrogen because of their chemistry and cage molecular structure, and the fullerene structure can easily be reversed back because of the higher C–C bond energies [3, 53, 54]. Conductive layers of carbon are applied on the electrode surface of super capacitors, and their capacitance relies on the surface area, pore size distribution, and electrical conductivity [55, 56]. Carbon-based nanomaterials provide higher electrical conductivity than orthodox available materials due to the higher surface area [57]. Fullerene-based composite materials have been reported to show higher specific capacitance of 135.36 Fg^− 1^ than the pure graphene material which was not hybridized with fullerene. In addition to this, fullerene-based composite exhibited better retention time rate of 92.35% even after a 1000 charge /discharge circle [58]. Fullerenes have also been utilized in lithium ion batteries as anode and provide better efficiency with replacement of nondegradable metallic anodes, thereby proving to be beneficial in terms of efficiency and an environmentally friendly material. The physicochemical properties of fullerenes also make them suitable candidates for the extraction of different species from the aqueous media [59, 60]. Pickering et al. designed water-soluble fullerene compounds and successfully applied them as sensitizer to produce reactive oxygen species (ROS) in water upon irradiation of visible and ultraviolet radiations. The ROS can photodegrade the organic contaminants in water, and in addition to this, the water-soluble fullerenes (fullerols) also act as anti-oxidants. Most importantly, fullerols can easily be removed from water after performing the function of photodegradation [53].

It is believed that fullerenes adsorbed species by the penetration of adsorbates in the spaces/defects between the carbon nanoclusters, and in addition to the defects, lower aggregation tendency and large surface area make them useful nanomaterials to be applied for adsorption of heavy metal ions from water [61, 62]. Alekseeva et al. conducted comparative studies of fullerene and nanocomposite-polystyrene film for the removal of Cu^2+^ ions; they found that fullerenes showed better efficiency [60]. They have also found that fullerenes follow the Langmuir model of adsorption for Cu^2+^ ions [60]. They established that the Cu^2+^ removal efficiency of fullerenes is higher in the first case, and the equilibrium isotherm of the Cu^2+^ adsorption on the fullerene fits the Langmuir model. Although fullerenes have great potential for water adsorption application, their cost is too high which restricts their utilization. However, the trace amount of fullerenes can be used to fabricate other materials like activated carbon, lignin, and zeolites to increase their efficiency of adsorption [63]. The fabrication of fullerene increases the hydrophobic character that makes materials better to be applied in adsorption and also helps in easier recycling [64]. Antibacterial material has been reported to be formed by grafting fullerene C_60_ with polyvinylpyrrolidone (PVP) which has the potential to be applied in water disinfection. Membrane technology is getting more and more attention in the purification of salts, organic matters, particles, and gases from water. Membrane performance depends on the composition of material as it is responsible for reactivity, selectivity, and mechanical strength. Fullerenes have strong potential to be applied in membrane technology because of their easy of functionalization, high electron affinity, great strength, ability to tailor size, etc. Fullerenes can be handy in grafting the nano-adsorbents to improve their adsorption efficiency.

#### Biocompatibility of Fullerenes

Fullerene-based nanomaterials have been exploited for biomedical application, and different studies have revealed their biocompatibility with living organisms. It has been used as a bioreceptor as well as a biosensor and also exploited in biomedical engineering, and it has been reported to be biocompatible with living systems [65–69]. The fullerene is a nontoxic material which can be exploited for filtration, adsorbents, and membrane stuff for environmental and water treatment applications.

### Carbon Nanotubes (CNTs)

Carbon nanotubes were discovered by Lijima in 1999, and they can be a single-wall (SWCNTs), e.g., layered rolled up graphene, or multiwall carbon nanotubes (MWCNTs), e.g., multilayered rolled up graphene [70]. The CNTs have been the focus of the nanotechnology research since their discovery due to their unique physicochemical properties. These cylindrical nanostructure allotropes of carbon are being applied in electronics, semiconductor, field emission, energy storage, catalysis, biomedical, air and water filters, etc. Their diameter can be of 1 nm to several nanometers with a large specific surface area (150–1500 m^2^/g), and they possess mesopores which make them the ideal candidate for the removal of heavy metal ions via adsorption [71, 72]. In addition to this, CNTs can easily be functionalized with various organic molecules which can make them specific for the selection of adsorbates and their adsorption capability could be improved [73]. The sorption mechanism of heavy metals by CNTs relies on the surface feature, electrochemical potential, and ion exchange process [11, 73].

#### Environmental Application of CNTs/CNT-Based Green Technology

There is an immense increase in global energy demand, and a lot of efforts are given to develop a simple, economical, and environmentally friendly material for reliable technologies for energy resource materials. Solar energy is produced by solar electric conversion and solar thermal conversion [74]. However, low efficiency of thermal conversion in solar collectors is the major hurdle.

Nanofluids of carbon are being widely applied in solar thermal conversion because of their good performance as solar absorbers [75]. Carbon nanotube-based photovoltaic cells (PVCs) got much attention of scientists as they can be p-type semiconductors with excellent mobility and their combination with electron donors is a new and unique idea [76, 77]. The organic photovoltaic devices can be easily designed by the fabrication of CNTs with polymers [78]. CNT -Si (p-n) heterojunction-based solar cells have been designed resulting in excellent conductive and transparent films. In addition to this, CNTs with n-type gallium arsenide (n-GaAs) has also been reported with better efficiency of 3.8% for green laser and desk lamp [79]. CNTs have also been widely exploited energy storage devices working on the principles of electrochemical double-layer capacitors (EDLCs) like ultracapacitors [80]. Incorporation of CNTs in electrodes of ultracapacitors resulted in much improvement in lifespan having more than 300,000 cycles [81]. In addition to this, super capacitor CNTs have also been utilized in diodes instead of conventional transistor as they can make perfect p-n junctions because of their excellent mechanical and electrical properties [58]. CNTs have also been widely utilized in advanced sensor technology as they can improve the sensitivity, selectivity, response time, cost-effectiveness, and lifetime of the chemical and biosensors [90]. These results suggest better CNTs to be ideal materials with excellent mobility and better efficiency with no negative impact on the environment which is a major drawback with most commonly applied metallic-based p-type materials.

##### Application of CNTs in Photocatalysis

Photocatalysis is one of the advanced technologies being applied for the wastewater treatment which utilizes semiconductors [82]. Variety of semiconductor materials are being applied namely Fe_3_O_4_, ZnO, and TiO_2_; however, quantum efficiency of these materials is not high, and in addition to this, their ultraviolet photo response is also slow [83]. CNTs are promising advanced materials for the catalysis because of their improved quantum efficiency, nano-size, high chemical stability, hollow tube structure, and extended light adsorption region due to their large specific surface area [84]. Gao et al. designed ultrathin network photocatalyst-based SWCNTs-TiO_2_ and successfully applied for the purification of water from oil [85]. Park et al. decorated titania on aerogel of SWCNTs and successfully applied for the removal of methylene blue from water [86]. Zhao et al. fabricated MWCNTs-TiO_2_ and applied for the photodegradation of methylene blue [87]. Xu et al. designed photocatalysts by the combination of hydroxy-MWCNTs and PbO_2_ nanocrystalline anode and applied it successfully for the removal of pyridine from water [88].

#### SWCNTs in the Purification of Heavy Metal-Contaminated Water

SWCNTs are one-dimensional (1-D) carbon nanomaterials made up of a hollow tube with walls being one atom thick. This 1-D material exhibits exceptional physicochemical properties due to its unique structure. SWCNTs are being widely applied in different fields such as semiconductors, electronics, biomedical sciences, chemical, and biosensors [44, 89–93]. SWCNTs are also widely used for the environmental pollution control because of their porous structure, high surface area, easier surface functionalization, and nanosize. These properties of SWCNTs are very promising for their application in water treatment. Alijani et al. designed SWCNT-based nanocomposite by fabricating them with magnetite cobalt sulfide, and resulting nanocomposites were applied for the removal of mercury; results showed high adsorption of more than 99.56% within a shorter period of 7 min [94]. In comparison to this, SWCNTs alone were found to adsorb 45.39% mercury [94]. Anitha et al. conducted a molecular dynamic simulation of bare SWCNTs and their functionalized counterparts, e.g., SWCNTs-OH, SWCNTs-NH_2_, and SWCNTs-COOH for the adsorption capacities of heavy metal ions, e.g., Cd^2+^, Cu^2+^, Pb^2+^, and Hg^2+^ from aqueous media. The results revealed that the SWCNTs-COOH have much adsorption capacities of about 150–230% higher compared to bare SWCNTs. The SWCNTs-OH and SWCNTs-NH were found to be weak in adsorption as they just showed 10–47% higher adsorption compared to SWCNTs [95]. SWCNTs-COOH have also been reported for the adsorption of Pb^2+^, Cu^2+^, and Cd^2+^ ions with adsorption capacity of 96.02, 77.00, and 55.89 mg/g, respectively. In comparison to this, non-functionalized SWCNTs were found to adsorb 33.55, 24.29, and 24.07 mg/g, for the Pb^2+,^ Cu^2+^, and Cd^2+^ ions respectively [96]. Zazouli et al. designed SWCNT nanocomposites by functionalization them with l-cysteine. They applied the designed nanocomposites for the removal of mercury from water. The adsorption efficiency of the designed SWCNTs-cysteine was found to be 95% [97]. Gupta et al. designed SWCNTs-polysulfone nanocomposite-based membrane and applied for the removal of heavy metals. Incorporation of SWCNTs resulted in a reduction in pore size of the membrane and smoother surface. The designed membrane was found to show high rejection capability for metal ions and removed 96.8% Cr^+ 6^, 87.6% As^+ 3^, and 94.2% Pb^+ 2^ ions. The membrane with no SWCNTs showed only 30.3%, 28.5%, and 28.3% rejection for Cr^+ 6^, As^+ 3^, and Pb^+ 2^ ions respectively. These results show the improvement in the efficiency of the membrane due to the incorporation of SWCNTs [98]. Dehghani et al. applied SWCNTs for the removal of Cr^+ 6^ ions from the water and evaluated the effect of different parameters, e.g., contact time, initial pH, and initial Cr^+ 6^ ion concentration on the adsorption capacity. It was observed that adsorption efficiency was depending on pH, maximum efficiency was found at pH 2.5, and adsorption follows the Langmuir isotherm model [99]. These studies suggested that the single-wall carbon nanotubes are suitable for the treatment of heavy metal-contaminated water.

#### MWCNTs in the Purification of Heavy Metal-Contaminated Water

**Fig. 4:**
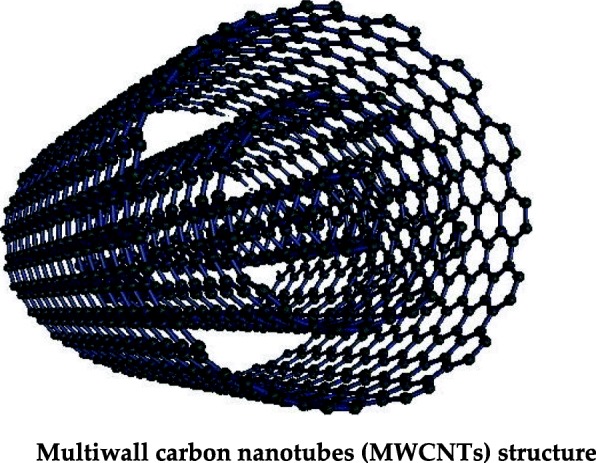
Multiwall carbon nanotube (MWCNT) structure

Carbon nanotubes having multiple rolled layers of graphene are called multiwall carbon nanotubes (MWCNTs), as shown in Fig. 4. The MWCNTs exhibit unique properties such as high surface area, high electrical, thermal conductivity, and high tensile strength [100]. Because of these physicochemical properties, they are widely applied in electronics, solar cells, sensors, and biomedical sciences [101–103]. The MWCNTs have also been widely applied in water treatment, and especially heavy metal ions are adsorbed by chemical interaction with functional groups of MWCNTs. The oxidized MWCNTs have been reported to have high adsorption capacity and efficiency for the Cr^6+^, Pb^2+^, and Cd^2+^ ions from the water [104, 105]. The metallic ion adsorption also depends on pH value, and this property can be applied for the desorption of ions by changing the pH, and MWCNTs can re-utilize. Some studies have revealed that plasma-oxidized MWCNTs have the better adsorption properties than chemically oxidized ones; this can be ascribed to higher number oxygenated functional groups present on the surface of the carbon nanotubes. Furthermore, it has been reported that plasma-oxidized MWCNTs can be easily recycled and reused [72, 106].

The composite material of MWCNTs has also been utilized for the adsorption of heavy metal ions from water. The MWCNTs-Fe_2_O_3_, MWCNTs-ZrO_2_, MWCNTs-Fe_3_O_4_, MWCNTs-Al_2_O_3_, and MWCNTs-MnO_2_-Fe_2_O_3_ nanocomposites have been successfully applied for the removal of the heavy ions of Cr^6+^, As^3+^, Ni^2+^, Pb^2+^, and Cu^2+^ ions from water [107–110]. The experimental conditions of solutions, including pH and metal ion concentrations, can affect the adsorption characteristics of MWCNTs, and the Freundlich adsorption model satisfied with their experimental data [81, 111]. The adsorption efficiency of functionalized MWCNTs increased comparative to other materials of organics oxides, and it is also predicted that functionalized MWCNTs are 20 times more effective in metal ion adsorption than unoxidized MWCNTs [112]. It is generally believed that links of ions and carbon nanotube polar surface occurrence are the main mechanism of sorption, [113, 114]. Oxidized MWCNTs have also shown exceptionally high sorption capacity and efficiency for Pb^2+^, Cd^2+^, and Cr^6+^ from the water. The sorption efficacy of MWCNTs with acid treatment increases the potential to remove lead, chromium, and cadmium ions with oxygen functional group making the complexes of ions or precipitates of salts on the surfaces [115]. Adsorption of MWCNTs treated with concentrated HNO_3_ increases significantly mainly due to oxygen functional groups created on the surface of acidified nanotubes that can react with metal ions to form complex or salt precipitates on the surface. The MWCNT composites with certain compounds like iron (III) oxide, zinc oxide, and aluminum oxide are formed by a coprecipitation method, and resulting composites are successfully applied for the removal of chromium, nickel, lead, copper, and arsenic ions. The adsorption efficiency of these nanocomposites was found to be dependent on the pH value and temperature, and the sorption process can be accomplished by changing these parameters [116, 117]. Depending on the pH and temperature, the sorption capacity of these composites varied from 10 to 31 mg/g. The adsorption process for these nanocomposites was well described by the Langmuir model [118]. The nanocomposites of oxidized multiwalled MWCNTs with manganese oxide/iron (III) oxide are reported to remove Cr^6+^ ions with maximum adsorption capacity of 186.9 mg/g with the maximum removal capacity of 85% at the optimum pH 2.1 studies. Their promising adsorption was due to the surface polarity of the adsorbents. It has also been reported that plasma-oxidized MWCNTs are better in adsorption compared to chemically oxidized ones as the prior ones have more oxygenated functional groups [119]. Plasma-oxidized technique has also been reported for the formation of nanotubes with titanium oxide and manganese dioxide and utilized for the removal of lead ions from water. The results showed that these hybrid systems can act as an effective adsorbent for the lead ions in the first case; the adsorption capacity was 137 mg/g, and in the second case, it was 78.74 mg/g [120]. In a heterogeneity adsorbent surface, sites combined twice are fitted in the isotherms models of the Langmuir-Freundlich equation that was used to differentiate between two types of adsorption sites with greater and lesser energy affinities for the Ni^2+^ ions [121]. It is believed that nickel ion sorption mainly occurs at the sites of energy with modified nanocomposites of MWCNTs and the nano-modification leads to a 20% increase in the adsorption capacity at small (up to 0.1 mol/l) equilibrium adsorbate concentrations. Another modification reported for MWCNTs is their functionalization with hydroxyquinoline and their application for the removal of copper, lead, cadmium, and other toxic ions [122]. The carbon nanotubes alone as well as in their oxidized and in their composite forms have tremendous ability to adsorb the heavy metal ions, and a lot of research is in progress for their applications in purification of water. Elsehly et al. applied commercial MWCNTs for the removal of the manganese and iron which could reach 71.5% and 52% respectively with a concentration in aqueous solution of 50 ppm of these metal ions [123]. In another study, CNT-based nanocomposites have been applied for the removal iron and manganese from the water [124].

##### Biocompatibility of CNTs

Carbon nanotubes have great potential to be applied for multidisciplinary fields like drug delivery, diagnosis, biosensors, electronics, semiconductors, and environmental remediations [125]. Different studies revealed the biocompatibility of CNTs as it has been widely exploited for biomedical applications [126, 127]. Carbon-based materials like CNTs are safe to be applied for the environmental remediation and in particularly for water treatment.

Graphene Based Material for Environmental Remediation

Graphene-based material for the adsorption of gaseous contaminants

Carbon dioxide (CO_2_) has been the environmental concern because of its immense effect in global warming [128]. Nanomaterials have been found to be promising materials as compared to conventional materials both with respect to cost and efficiency [129]. Graphene-based materials have been utilized for the adsorption of gaseous contaminants [130]. Gosh et al. showed the successful application of graphene-based nanomaterials for the capture of CO_2_ and H_2_. A single layer of graphene has been reported to capture 37.93% of CO_2_ [130]. Graphene has been reported to selectively adsorb CO_2_ as compared to methane (CH_4_) and nitrogen (N_2_) gases. Selectivity of graphene oxide (GO) for CO_2_ can be attributed to higher dipole moment of carbon dioxide which can easily interact with polar oxygenated functional groups of CO_2_ [74, 131]. Other studies have also been reported for tuning the graphene chemistry for the improved selectivity of the desired gaseous contaminant [75, 131].

Graphene Oxide in Removal of Organic Dyes from Water

Graphene-based nano-adsorbents are excellent advanced materials for the removal of the organic contaminants from the water because of their nano-scaled size, high surface area, ability to interact via pi-pi stacking, hydrogen bonding, and electrostatic interactions [26]. In comparative adsorption studies of GO and graphite using methylene blue and malachite green as standard organic dyes, it was found that GO showed much better adsorption than graphite [26]. GO has also been utilized for the removal of cationic dyes namely methylene blue (MB), crystal violet (CV), and rhodamine B (RhB) from water. It was found that the higher the initial dye concentration, the higher will be the adsorption with adsorption capacities of 199.2, 195.4, and 154.8 mg g^−1^ for MB, CV, and RhB, respectively [76]. GO has also successfully applied for the removal of anionic dyes like Acid Orange 8 (AO8) and Direct Red 23 (DR23) from aqueous solutions [77].

Graphene-Based Photocatalytic Materials for Water Decontamination

Although adsorption can remove the contaminant from water, the adsorption technique is unable to destroy/degrade the contaminants and disposal step is required [77]. Photocatalysis is a useful approach for water remediation/wastewater treatment for the complete degradation and mineralization of organic/biological contaminants [78]. Graphene-based photocatalysts have been reported for their improved activity because of their high surface area, nanosize, and more electronic movements as compared to the traditionally used materials [78, 132]. Rommozzi et al. designed reduced graphene oxide (rGO) with a greener reduction method using glucose and ammonium hydroxide and successfully designed a photocatalyst which is visible by the fabrication with TiO_2_. The designed rGO-TiO2 photocatalyst was successfully applied for the refractory dye named Alizarin Red S (ARS) [133]. In other studies, graphene oxide fabricated with TiO_2_ and ZnO exhibited much photodegradation of methylene blue as compared to TiO_2_/ZnO alone [79, 80].

### Graphene and Graphene Oxide-Based Adsorbents for the Purification of Heavy Metal-Contaminated Water

**Fig. 5:**
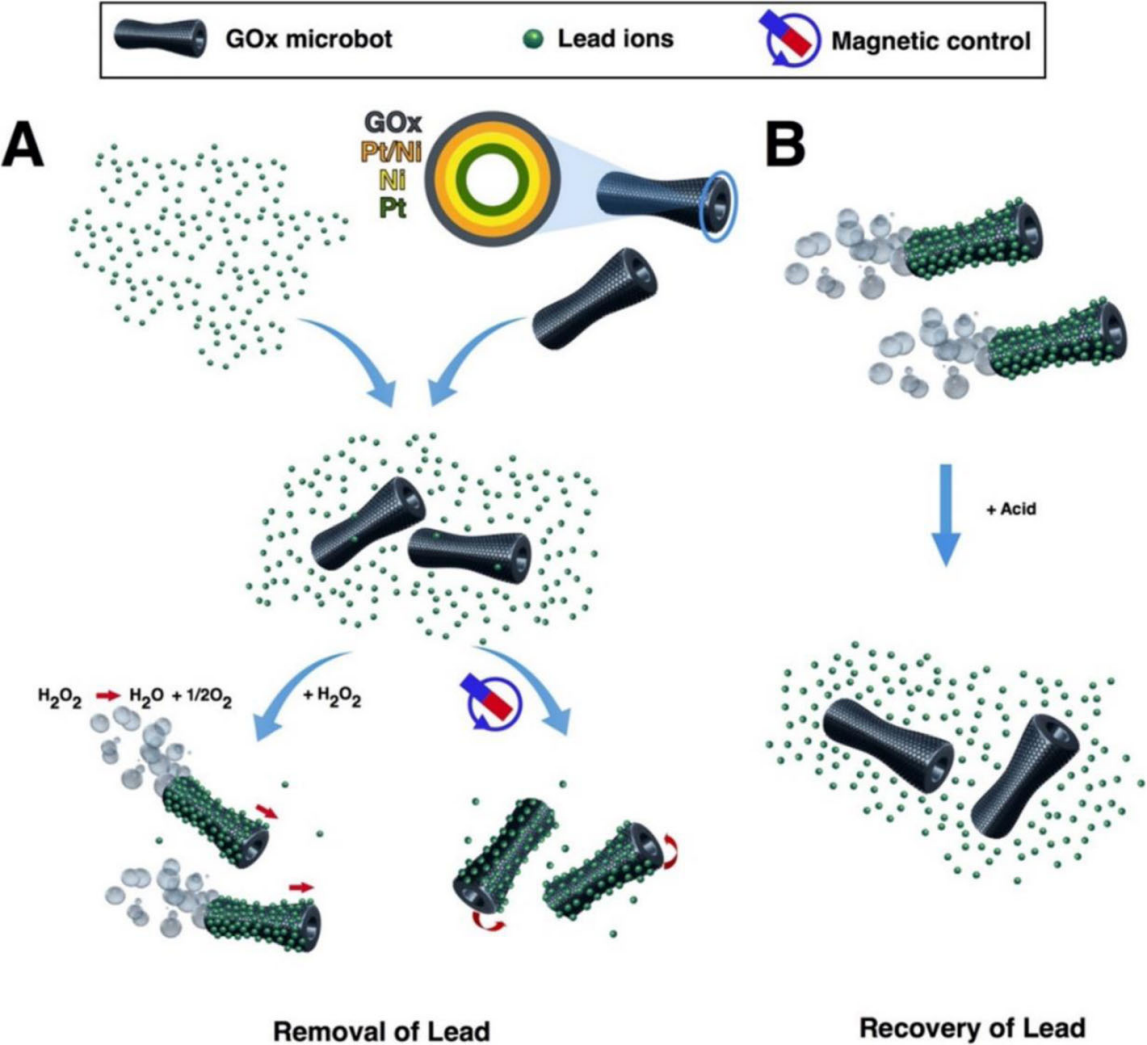
Scheme of GOx-microbot-based approach for lead decontamination and recovery. **a** Decontamination of polluted water using GOx-microbots fabricated by electrodeposition of nanolayers of graphene oxide (GOx), Pt/Ni layer, Ni magnetic layer, and Pt catalytic inner layer. The decontamination strategy for lead ions can be carried out by two different techniques: self-propulsion of the GOx-microbots in the presence of H_2_O_2_ or by using an external rotating magnetic field. **b** Recovery of lead ions from the GOx-microbots in the presence of acidic media [117]

Graphene is one-atom-thick-layered hexagonal lattice of carbon atoms and is known as the thinnest material with the strength of 200 times than steel. Graphene was discovered in 2004 by Sir Andre Geim and Sir Konstantin Novoselov, who were awarded a Nobel prize for their discovery in 2010. Graphene (2-D) is being used widely in almost every field such as in touch screens, mobiles, LCDs, semiconductors, computer chips, batteries, energy generation, water filters, supercapacitors, solar cells, and biomedical and environmental sciences [134–137]. These 2-D graphene-based materials are getting more and more attention in water treatment due to their unique physicochemical characteristics namely electronic properties, high surface area, thermal mobility, high mechanical strength, and tunable surface chemistry [118, 134, 138, 139]. Tabish et al. designed porous graphene and applied it as an adsorbent for the removal of heavy metal ions as well as other pollutants from water. They applied this porous graphene material for As^3+^ removal from water and found 80% efficiency. The material was found to retain its water treatment properties after regeneration and recycling [138]. Guo et al. designed a nanocomposite of partially reduced graphene oxide by its fabrication with Fe_3_O_4_ via in situ co-precipitation method and applied it for the removal of Pb^2+^ ions from water. The designed nanocomposite was found to be excellent in removing the Pb^2+^ ions from aqueous solution with an adsorption capacity of 373.14 mg/g [140]. Zhang et al. functionalized the reduced graphene oxide with 4-sulfophenylazo (rGOs) and applied it for the removal of a variety of heavy metal ions from aqueous solution. The designed material showed the maximum adsorption capacity of 689, 59, 66, 267, and 191 mg/g for the Pb^2+^, Cu^2+^, Ni^2+^, Cd^2+^, and Cr^3+^ respectively [141]. Diana et al. designed a graphene-based self-propelled microbot system whose structure was made up of nanosized multilayered consisting of graphene oxide, nickel, and platinum. Each layer performed a different function, e.g., graphene oxide captures the heavy metal Pb^2+^ ions, the middle layer of Ni enables the control of microbots with the help of external magnetic field, and the inner layer of platinum helps the engine in self-propelling [142]. The designed system was found to remove the 80% of the Pb^2+^ water solution. Figure 5 shows the schematic illustration of the working principle of microbots. Yang et al. designed hydrogen beads using graphene oxide and sodium alginate (GO-SA) and successfully applied them for the removal of Mn^2+^ ions from the aqueous solution with excellent adsorption capacity of 56.49 mg/g [9]. Zheng et al. designed nanocomposites by fabrication of zinc oxide with tea polyphenol with reduced graphene oxide (TPG-ZnO). Designed material was applied for the removal of heavy metal ions with an added advantage of antibacterial properties [143]. They applied this material for the removal of Pb^2+^ ions from aqueous solution with adsorption efficiency of 98.9%, and the adsorbent was found to possess antibacterial properties against *Streptococcus mutans* with 99% eradication [143]. Mousavi et al. designed nanocomposites of graphene oxide with iron oxide magnetite nanoparticles Fe_3_O_4_ and applied them for the removal of Pb^2+^ ions from water and the material showed 98% removal efficiency with a capacity of 126.6 mg/g [144]. Considering functionalized graphene as an adsorbent to remove Pb^2+^ ions from an aqueous medium, the highest record of Pb^2+^ ion removal over graphene is 406.6 mg/g at pH of 5.0 in 40 min [145]. Graphene-hydrogel lingo sulfonate functionalized nanocomposites having oxygenated functional groups making the surface highly polar reported to increase the rate of adsorption of Pb^2+^ ions with maximum efficacy of 1308 mg/g with the equilibrium reached in 40 min. Awad et al. modified graphene oxide with chloroacetic acid (GO-COOH) and ethylenediamine (GO-amino). The designed systems were applied for the removal of mercury (Hg^2+^) from water and found that the nanocomposites (GO-COOH) and (GO-amino) have an adsorption capacity of 122 mg/g and 230 mg/g. In addition to this, designed systems retained their adsorption efficiency after the recycling process [146]. Yan et al. designed magnet graphene oxide for the rapid removal and separation of Fe (II) and Mn (II) from micropolluted water [147]. Ali et al. designed graphene-based adsorbent successfully for the removal of noxious pollutants namely Cu (II), Pb (II), Fe (II), and Mn (II) [148].

#### Biocompatibility of Graphene-Based Nanomaterial

Graphene-based nanomaterials have been applied in different fields like electronics, chemical sensors, biosensors, drug delivery, theragnostic, and other related biomedical fields. These studies also report the cytocompatibility of graphene-based materials both by in vitro as well as in vivo animal studies [122, 133, 149–152]. These findings suggest that the graphene-based materials are safe for the environmental remediation application as they are just used for the removal and degradation of pollutants and are not consumed by humans directly.

#### Activated Carbon in Environmental Remediation

Activated carbon is a fabulous material because of its high surface area, highly porous structure, and ease of preparation with variety of starting materials. Because of its ideal physicochemical properties, it has wide application in environmental remediations in different industries like pharmaceutics, fertilizer plants, petroleum, cosmetics, automobiles, and textiles [153] It is also widely applied for the adsorption of gases, solvent recovery, and wastewater treatment especially for the removal of organic dyes/other pollutants; not only this, but it is also used as a catalyst in biodiesel production. It is also applied as a low-cost material for the treatment and removal of water containing COD, BOD, and TSS and stabilizing and maintaining the optimum pH for biological uses [154–156]. Maguana et al. prepared activated carbon from the pear seed cake and successfully applied it for the removal of methylene having an adsorption capacity of 260 mg/g [157]. Antonio et al. prepared activated carbon from the kenaf plant and applied it successfully for the treatment of the wastewater of hospitals containing paracetamol as the main pollutant [158]. The above literature suggests that the activated carbon is the pretty useful economical material which can easily be prepared and it has immense application in environmental remediations.

### Activated Carbon as Adsorbents in the Purification of Heavy Metal-Contaminated Water

**Fig. 6:**
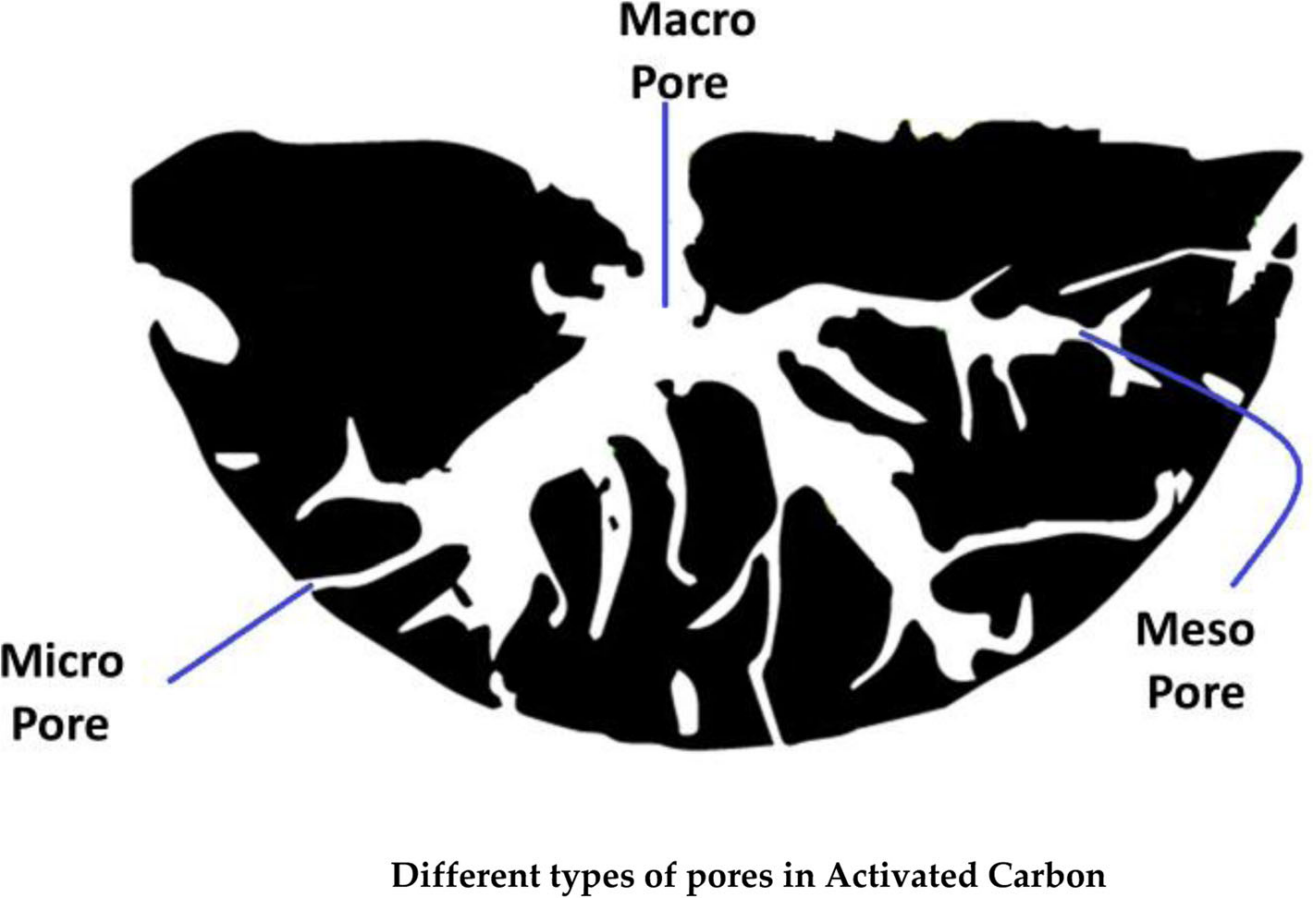
Different types of pores in activated carbon

Activated carbon (AC) is also known as activated charcoal, and this of type carbon material is formed under some treatment protocols resulting in micro/nanopores and having the large surface area of more than 3000 m^2^ [159]. The AC is produced on a large scale from coal, wood, and agricultural wastes [160]. In addition to its porous nature (as shown in Fig. 6), AC also has a high mechanical strength which enables its applications in catalyst support, capacitors, electrodes, and gas storage and most importantly used as the adsorbent for removal of metal ions, organic wastes, and gases from water [160–162]. The high mechanical strength of activated carbon enables its periodic cleaning, regeneration, and reutilization [160]. Abeer et al. reported the preparation of AC from apricot stone and its application in removal of Zn^+ 2^ and Al^+ 3^ ions with removal efficiency of 92% [163]. Ebrahim et al. designed AC from sewage sludge, applied it for the removal of Cu^+ 2^ ions from water, and found that the designed material showed maximum adsorption capacity of above 50% [164].

Li et al. prepared the AC from sewage sludge produced from the wastewater treatment plant and functionalized it with sulfur [165]. They applied sulfonated AC for the removal of Pb^2+^, Cd^2+^, Cu^2+^, and Ni^2+^ ions from water. The adsorption capacity of metal ions were found to be 238.1 mg/g, 96.2 mg/g, 87.7 mg/g, and 52.4 mg/g for Pb^2+^, Cd^2+^, Cu^2+^, and Ni^2+^ respectively [165]. Cao et al. designed multipore activated carbon (MPAC) with a large surface area using the agricultural waste of long-root *Eichhornia crassipes* and applied it for removing heavy metal ions, e.g., Pb^2+^, Cd^2+^, Cu^2+^, Ni^2+^, and Zn^2+^. They found that at 30 °C adsorption capacity being 1.34 mmol/g, 1.07 mmol/g, 1.22 mmol/g, 0.97 mmol/g, and 0.93 mmol/g for Pb^2+^, Cd^2+^, Cu^2+^, and Ni^2+^ respectively [166]. Dong et al. investigated the application of spent activated carbon (AC) for heavy metal ion removal from water and found high adsorption capacity of 95% and 86% for Pb^2+^ and Cd^2+^ ions respectively [167]. M. Bali et al. [168] applied commercial AC for the removal of heavy metal ions and found that adsorption equilibrium of Cd^2+^ ion took 15 min while for Pb^2+^, Zn^2+^, and Cu^2+^ it took 45 min with percentage removal of 64% for all these ions and with Cd^2+^ being the highest [10]. Kongsuwan et al. prepared the activated carbon from the agricultural waste of eucalyptus bark. They applied it for the removal of Cu^2+^ and Pb^2+^ from water with maximum removal capacity of 0.45 and 0.53 mmol per gram of AC respectively, with adsorption being the main mechanism of ion uptake [169]. AC poultry litter has also been reported and applied for the treatment of heavy metal-contaminated water and found that for 1 kg of poultry litter AC adsorbs 404 mmol, 945 mmol, 236 mmol, and 250–300 mmol of Cu^2+^, Pb^2+,^ Zn^2+^, and Cd^2+^ ions respectively [170]. This adsorption is significantly higher than the commercially available AC derived from coconut and bituminous. The AC of wood saw dust of rubber plant has also been reported for the removal of heavy metal ions of Cr^+ 6^ from water with adsorption capacity of 44 mg/g [171]. AC formed from Moso and Ma bamboo was found to be highly efficient in removing the heavy metal ions, i.e., Pb^2+,^ Cu^2+^, Cr^3+^, and Cd^2+^ with the maximum adsorption capacity of more than 90% removal [172]. Naser et al. prepared AC from rice husk and applied them for the removal of Cu^2+^ from aqueous solution, and maximum capacity was found to be 33.92%. Similar results have also been reported for the removal of Cu^2+^ from the AC formed from Palm shell [173]. AC of love stones has been reported for the adsorption of Cd^+ 2^ and Ni^+ 2^ with adsorption capacity of 1.85 mg/g and 0.67 mg/g respectively in two different studies [174, 175]. AC prepared from olive stone using the microwave method has been applied for the removal of Fe^2+,^ Pb^2+,^ Cu^2+,^ Zn^2+,^ Ni^2+^, and Cd^2+.^from wastewater. Another study reported on the olive stone AC prepared via microwave to remove a group of metal ions from synthetic wastewater: Fe^2+^, Pb^2+^, Cu^2+,^ Zn^2+^, Ni^2+^, and Cd^2+^ with removal efficiency of more than 98% [176]. Tamarind wood AC has been reported for the highest adsorption capacity of above 97% for Pb^2+^ from water [177]. Activated carbon has been applied as an adsorbent for the removal of Fe (II) and Mn (II) with great efficiency [178, 179]. The activated carbon is easy to synthesize, is cheaper, and is the most promising material for the adsorption of heavy metal ions and can be prepared at a large scale from a variety of carbon sources especially form agricultural waste. In addition to easier preparation, AC can easily be functionalized. Table 2 summarizes the effect of different parameters on the process of metal ion adsorption.

**Table 2:** Effect of different parameters on the process of metal ion adsorption

S. No	Adsorbent	Metal ions	Contact time in hours	Optimum pH for adsorption	Adsorption capacity (mg/g)/efficiency (%)	Reference
1	SWCNTs	Hg^2+^	1.50	7.94	41.66 mg/g	Alijani et al. 2018 [72]
2	SWCNTs-Fe_3_O_4_-CoS	Hg^2+^	0.11	5.26	1666 mg/g	Alijani et al. 2018 [72]
3	SWCNTs-polysulphone (membrane)	Cr ^6+^	Flux through membrane	2.6	96.8%	Gupta et al. 2015 [84]
		As ^3+^			87.6%	
		Pb^2+^			94.2%	
4	SWCNTs	Cr^+6^	1.00	2.5	2.35 mg/g	Dehghani et al. 2015 [85]
5	MWCNTs	Cr^+6^	1.00	2.5	1.26 mg/g	Dehghani et al. 2015 [85]
6	Functionalized MWCNTs	Pb^2+^	6.00	9.0	93%	Farghali et al. 2017 [91]
		Ni^2+^			83%	
		Cu^2+^			78%	
		Cd^2+^			15%	
7	Functionalized MWCNTs	Cr^3+^	3.00	6.0	99.83%	Ahmad et al. 2015 [102]
8	Al_2_O_3_-MWCNTs	Pb^2+^	1.00	7.0	90%	Gupta et al. 2011 [103]
9	Fullerene (C6)	Cu^2+^	0.33	Not monitored	14.6 mmol/g	Alekseeva et al. 2016 [58]
10	Porous graphene	As ^3+^	1.00	7.0	90%	Tabish et al2018 [67]
11	rGO-Fe_3_O_4_	Pb^2+^	0.16	6.0	373.14 mg/g	Guo et al. 2018 [69]
12	Reduced GO-sulfophenylazo (rGOS)	Pb^2+^	0.16	5.0	689 mg/g	Zhang et al.2018 [116]
		Cu^2+^			59 mg/g	
		Ni^2+^			66 mg/g	
		Cd^2+^			267 mg/g	
		Cr^3+^			191 mg/g	
13	Tea polyphenols—rGO-ZnO	Pb^2+^	1.83	7.0	98.9%	Zheng et al. 2018 [118]
14	GO-Fe_3_O_4_	Pb^2+^	Not mentioned	6.1	126.6 mg/g	Mousavi et al. 2018 [119]
15	GO-iminodiacetic acid	Hg^2+^	3.33	5.0	230 mg/g	Awad et al.2018 [121]
16	GO-COOH	Hg^2+^	0.83	5.0	122 mg/g	Awad et al.2018 [121]
17	Activated carbon (from apricone)	Al^3+^	2.00	6.0	92.86%	Abeer et al. 2018 [126]
		Zn^2+^		6.5	93.88%	
18	Activated carbon (sewage sludge)	Cu^2+^	12.00	Not mentioned	50.6%	Ibrahim et al. 2018 [127]
19	Activated carbon (sewage sludge)	Pb^2+^, Cd^2+^, Cu^2+^, Ni^2+^	24	5.0	238.1 mg/g, 96.2 mg/g, 87.7 mg/g 52.4 mg/g	Li et al. 2018 [128]
20	Multipore activated carbon (long-root *Eichhornia crassipes*)	Pb^2+^, Cd^2+^, Cu^2+^, Ni^2+,^ Zn^2+^	2.50	6.0		Cao et al.2019 [129]
21	Spent activated carbon (AC)	Pb^2^ Cd^2+^	2.00	8.0	95%	Dong et al. 2018 [130]
					86%	
22	Commercial activated carbon	Cd^2+^ Pb^2+^, Zn^2+^ Cu^2+^	0.25	Not mentioned	78.43%	M. Bali et al. 2018 [168]
			0.75		64.75%	
			0.75		70.77%	
			0.75		67.07	

#### Biocompatibility of the Activated Carbon

Different studies have been conducted for the biocompatibility evaluation of the activated carbon materials prepared form different carbon sources. Activated carbon has been applied for the treatment of cystitis and was found to be effective and nontoxic compared to the antibiotics being applied [180]. Biocompatibility of activated carbon can be attributed to its inertness, and it has also been functionalized and fabricated with other materials to confer on the disinfection properties [181]. The activated carbon is also given orally to human beings as a sorbent for the removal of toxins from the human body and has also been utilized in biomedical applications [182, 183]. These studies strongly suggest the biocompatibility of the activated carbon.

## Conclusion

In this review, environmental and special purification of heavy metal from heavy metal contaminants by the applications of carbon nanomaterials, namely fullerene carbon nanotubes, graphene, graphene oxide, and activated carbon discussed. These carbon nanomaterials have been utilized in the purification of heavy metal-contaminated water with great success. The reason behind the successful application is due to their fascinating properties like high surface area, ease of recycling, and easiness to desorb the adsorbed metal ions; only using mineral acid solution and regenerated material can be reused with retention of adsorption capability. In addition to these properties, the carbon nanomaterials can easily be fabricated with other nanomaterials and are easy to be functionalized resulting in multifunctional nano-adsorbent. Carbon-based materials are highly biocompatible with living organisms and environment. There is also an immense effect of different parameters such as pH, contact time, and type of adsorbents on the process of metal ion adsorption. Based on this literature review, it can be concluded that carbon nanomaterials have fascinating physicochemical properties and have great potential to be exploited in the environmental remediation and water purification.

## Data Availability

All data are fully available without restriction.

## Abbreviations

AC: Activated carbon
GO: Graphene oxide
MWCNTs: Multiwall carbon nanotubes
r-GO: Reduced graphene oxide
SWCNTs: Single-wall carbon nanotubes
